# The relative influences of product volume, delivery format and alcohol concentration on dry-time and efficacy of alcohol-based hand rubs

**DOI:** 10.1186/1471-2334-14-511

**Published:** 2014-09-20

**Authors:** David R Macinga, David J Shumaker, Heinz-Peter Werner, Sarah L Edmonds, Rachel A Leslie, Albert E Parker, James W Arbogast

**Affiliations:** GOJO Industries, Inc, One GOJO Plaza, Suite 500, Akron, OH 44311 USA; Department of Integrative Medical Sciences, Northeastern Ohio Medical University, Rootstown, Ohio 44272 USA; HygCen International GmbH, Werksgelände 24, Bischofshofen, 5500 Austria; Center for Biofilm Engineering at Montana State University, Bozeman, Montana 59717 USA; Department of Mathematical Sciences at Montana State University, Bozeman, Montana 59717 USA

**Keywords:** Alcohol-based hand rub, ABHR foam, Application volume, Dry-time, *In vivo* efficacy, EN 1500

## Abstract

**Background:**

Alcohol-based hand rubs (ABHR) range in alcohol concentration from 60-95% and are available in a variety of delivery formats, such as rinses, gels, and foams. Recent studies suggest that some ABHR foams dry too slowly, thereby encouraging the use of inadequate volumes. This study investigates the influence of product volume, delivery format, and alcohol concentration on dry-time and antimicrobial efficacy of ABHR foams, gels and rinses.

**Methods:**

ABHR dry-times were measured using volunteers to determine the influences of product volume, delivery format, and alcohol concentration. ABHR efficacies were evaluated according to the European Standard for Hygienic Hand Disinfection (EN 1500) using 3-mL application volumes rubbed for 30 s, and additionally, using volumes of the products determined to rub dry in 30 s.

**Results:**

Volumes of six ABHR determined to rub dry in 30 s ranged from 1.7 mL to 2.1 mL, and the rate of drying varied significantly between products. ABHR dry-times increased linearly with application volume and decreased linearly with increasing alcohol concentration, but were not significantly influenced by product format. An ABHR foam (70% EtOH), rinse (80% EtOH), and gel (90% EtOH) each met EN 1500 efficacy requirements when tested at a volume of 3 mL, but failed when tested at volumes that dried in 30 s.

**Conclusions:**

Application volume is the primary driver of ABHR dry-time and efficacy, whereas delivery format does not significantly influence either. Although products with greater alcohol concentration dry more quickly, volumes required to meet EN 1500 can take longer than 30 s to dry, even when alcohol concentration is as high as 90%. Future studies are needed to better understand application volumes actually used by healthcare workers in practice, and to understand the clinical efficacy of ABHR at such volumes.

**Electronic supplementary material:**

The online version of this article (doi:10.1186/1471-2334-14-511) contains supplementary material, which is available to authorized users.

## Background

Alcohol-based hand rubs (ABHRs) are recommended for routine hand disinfection in healthcare settings when hands are not visibly soiled [1, 2]. Their role and importance has been highlighted by numerous studies associating their use with clinical reductions in hospital-acquired infections (HAIs) [1, 2]. ABHR are available in a number of different delivery formats, such as rinses (i.e., thin liquids), gels, and foams. Each product format may offer a unique functional benefit, depending on the situation. For example, because gels and foams tend to drip from the hands less than rinses, the entire volume is delivered to the hands. And, beyond their functional attributes, there can be aesthetic preferences for specific ABHR formats [3].

Despite a large body of evidence supporting the use of ABHR for infection prevention, consensus is lacking as to the appropriate amount of a product that should be applied to the hands. The US Centers for Disease Control and Prevention (CDC) Guidelines for Hand Hygiene in Healthcare Settings acknowledges that the ideal volume of product to apply to the hands is unknown, but states that, if hands dry before 10 to 15 s, an insufficient amount was used [1]. The World Health Organization (WHO) Guidelines on Hand Hygiene in Health Care recommends that a “palmful” of product be used and states that the hand-hygiene event should take 20-30 s [2]. Despite lack of specific guidance on application volume, it has been clearly demonstrated by *in*-*vivo* microbiological studies and in clinical settings that antimicrobial efficacy of ABHR is directly proportional to application volume, and that ABHR will not meet global efficacy standards if insufficient volumes of product are applied [4–7]. It should be noted, further, that success criteria for current efficacy standards are arbitrary and not based on clinical outcomes [8, 9].

A recent study by Kampf *et al.* found that recommended use volumes for ABHR foams containing 62% ethanol exceeded the WHO recommended dry-time of 30 s, and that a volume that rubbed dry in 30 s (1.6 mL) was insufficient to meet the efficacy criterion of EN 1500 [10]. The authors concluded that because the “…time required for dryness often exceeds the recommended 30 s … only a small volume of these foams (below that required to meet efficacy norms) is likely to be applied in clinical practice”. The study evaluated only ABHR foams, and all test products contained the same alcohol concentration; therefore, broad conclusions should not be drawn as to whether the observed drying rates are attributable to product format or to alcohol concentration. A study by Rotter *et al.* found that 3 mL of the EN 1500 Reference Product required, on average, more than 49 s to dry, despite the method specifying a rub-time of 30 s [11]. These data suggest that dry-time versus volume of an ABHR, regardless of delivery format, is an important relationship for study.

The objectives of this study were 1) to determine the influence of product volume, delivery format, and alcohol concentration on ABHR dry-time and antimicrobial efficacy, and 2) to investigate the efficacy of ABHRs when applied at volumes drying in 30 s, as specified by WHO.

## Methods

### Test Products

The identities of the test products used in this study, listed in Table 1, were blinded to the test subjects. Product densities, measured using an Anton Paar DMA 4500 Density Meter at 60°F, were used to convert ABHR mass to volume.

**Table 1:** **Products tested in this study**

Code	Test product name	Manufacturer	Active ingredient	Density (g/mL)
Foam A	PURELL® Instant Hand Sanitizer Foam	GOJO Industries	62% ethanol (v/v)	0.8940
Foam B	PURELL® Advanced Instant Hand Sanitizer Foam	GOJO Industries	70% ethanol (v/v)	0.8739
Gel C	PURELL® Advanced Instant Hand Sanitizer	GOJO Industries	70% ethanol (v/v)	0.8739
Rinse D	Experimental Prototype	GOJO Industries	70% ethanol (v/v)	0.8739
Rinse E	WHO-recommended hand rub formulation with ethanol	n/a	80% ethanol (v/v)	0.8661
Gel F	Sterillium® Comfort Gel™	Bode Chemie	90% ethanol (v/v)^a^	0.8331
			[85% ethanol (w/w)]	

^a^Ethanol concentration on product label is reported as weight per weight (w/w); (v/v) concentration was determined analytically in the authors’ laboratory (See Methods).

### Determination of ABHR dry-times

ABHR gels, rinses, and ethanol-in-water solutions were applied using adjustable pipettes set to a desired volume. For gels, positive-displacement pipettes were used. ABHR foams were dispensed from pumps delivering a known constant quantity (0.4 mL), and total volumes were controlled by the number of pump activations. A specified quantity of an ABHR was placed in a subject’s cupped palms, and the subject rubbed the product onto all surfaces of the hands up to the wrists “until the hands felt dry” to them. No instructions were given regarding rub-in technique. A calibrated digital timer was used to record the time interval from when a subject began rubbing to when they indicated their hands felt dry. After using each test product, subjects washed their hands with a bland soap and waited at least 30 minutes before a subsequent evaluation. Ethical approval was not considered necessary for this part of the study.

### Comparison of ABHR drying rates among multiple products

Dry-times for six ABHRs representing different product formats, and ranging in ethanol concentration from 62% to 90% (Table 2), were measured across a range of application volumes. A panel of thirteen subjects evaluated multiple volumes of the ABHR products in random order, a single product per test day. Mean dry-times recorded from all subjects were plotted against the product volumes used and analyzed by linear regression (GraphPad Prism 5.04, GraphPad Software, Inc., San Diego, CA) to determine the rate of drying (s/mL) and the volume of each product that should dry in 30 s. Because variability of the dry-times tended to increase with increased volume (i.e., demonstrated heteroscedasticity), a weighted regression model with fixed effects for Product and the Product-Volume interaction was used to compare dry-time rates as volume of product increased. To account for the repeated measures from each subject, the model also included a random intercept and rate for subject in the free statistical and graphing program R [12, 13]. Residual and normal probability plots were used to confirm that heteroscedasticity of variance was adequately modeled. To maintain a family-wise false discovery rate of 5% among the follow-up *t* tests, a Benjamini-Hochberg correction was applied.

**Table 2:** **Statistical comparison of drying rates and volumes of six ABHR test products as a function of volume**

Test product	Active ingredient	Volume in mL drying in 30 s^a^	Drying rate in s/mL (95%CI)^b^	Drying rate Significance groups^c^				
Foam A	62% ethanol (v/v)	1.7	18.2 (15.5-21.0)	1				
Foam B	70% ethanol (v/v)	1.7	17.2 (14.5-19.8)	1	2			
Rinse D	70% ethanol (v/v)	1.7	15.5 (12.9- 18.0)		2	3		
Gel C	70% ethanol (v/v)	1.9	14.0 (11.5-16.4)			3	4	
Rinse E	80% ethanol (v/v)	2.0	12.8 (10.4-15.2)				4	5
Gel F	90% ethanol (v/v)^d^	2.1	12.2 (9.8-14.7)					5

^a^Determined from the simple linear regression analysis where T = 30 s.

^b^Change in dry-time per mL determined by a weighted regression model (see Methods).

^c^Test products with the same number are not significantly different (See Methods).

^d^Concentration on product label is reported as weight per weight (w/w); (v/v) concentration was determined analytically in the authors’ laboratory (See Methods).

### Relationship between ABHR dry-time and product delivery format

To evaluate the specific influence of delivery format (gel, rinse, or foam) on dry-time, a panel of nurses evaluated each of three closely related test products (i.e., having identical ethanol concentrations and nearly identical excipient ingredient composition) delivered at two specific volumes, 0.8 mL and 1.6 mL, estimated to dry in approximately 15 s and 30 s, respectively, based on data for application volume versus dry-time. The sample size (*n* = 30) was calculated specifically to enable detection of a difference in dry-times as small as 5 s at 95% power. The calculation was based on data from the testing of dry-times versus application volume using thirteen subjects (Table 2). Rinse D represented the base formulation, with Foam B differing only by addition of a foaming agent (<2% w/w), and Gel C by addition of a gelling agent (<0.3% w/w). Each formulation comprised more than 97% alcohol and water w/w, with non-volatile ingredients contributing less than 3% w/w. In addition to recording dry-times, occurrence of product dripping from a subject’s hands during application and rubbing was noted. These data were analyzed using an ANOVA (and fit in R) that accounted for the repeated measures from each subject by including a random effect for subject and fixed effects, including the two-way interaction, for product format and dripping events. From this ANOVA, three Bonferroni simultaneous 90% confidence intervals (CIs) were generated to compare the mean dry-times amongst the three product formats, with statistical equivalence at 95% confidence concluded if all three Cls were contained in the interval (-5, 5) [14]. In other words, mean differences as large as 5 seconds were assumed negligible and not of practical importance.

### Relationship between ABHR dry-time and ethanol concentration

In a separate, but related experiment a panel of eleven subjects was used to determine dry-times for 1.7-mL volumes of five ethanol-in-water rinse solutions ranging in concentration from 50% to 90% w/w in 10% increments. The 1.7-mL volume was selected based on data from the testing of dry-times versus application volume, viz., the volume of the two in-test ABHR foams that rubbed dry in 30 s (Table 2). A repeated-measures regression analysis with a random intercept and rate for subject was applied to determine the linear relationship between ethanol concentration and ABHR dry-time (in R). A repeated-measures ANOVA (Minitab 16) was used to compare the mean dry-times amongst the five ethanol concentrations used in the study, with a random effect for subject.

### *In-vivo*evaluation of antimicrobial efficacy

The influence of application volume on ABHR efficacy was evaluated in two studies conducted according to the CEN phase 2/step 2 standard EN 1500 rev:2009 (Hygienic Handrub) methodology, as described previously [8, 15]. Studies were conducted in Germany and were performed in compliance with the *Declaration of Helsinki (DoH): Ethical Principles for Medical Research Involving Human Subjects* (October 2013). Ethics board approval was not required based on the classification of *Escherichia coli* K12 (NCTC 10538) as a Risk Group 1 non-pathogenic organism by the German Safety Ordinance on Gene Technology. The laboratory was accredited in accordance with EN ISO 17025 (EN 45000) and recognized by ZLG, German Central Authority of the federal states for Health Protection of Pharmaceuticals and Medical Products. All areas of testing were approved and reported to the Ministry of Labour, Gender and Social Affairs of the Federal State of Mecklenburg - Western Pomerania, Germany. Written informed consent was obtained from each subject prior to their participation in the study. A Latin-square design was used in each study to test three test products and a reference product (60% isopropyl alcohol), with 20 subjects per study for a total of 40 subjects. The reference product was evaluated as specified in the standard: 3 mL rubbed for 30 s, followed by an additional 3 mL rubbed for 30 s. Test products were evaluated by each subject using a single application of each product. In the first study, test products were evaluated at an application volume of 3 mL and rubbed for a timed 30 s, in accordance with EN 1500 rev:2009 procedure, after which the fingertips were rinsed for 5 s under running tap water, excess water shaken off, and hands immediately sampled. In the second study, the procedure was modified such that test products were rubbed until completely dry and then, without rinsing, sampled immediately thereafter. Neutraliser (3.0% polysorbate 80 + 3.0% saponine + 0.1% histidine + 0.1% cysteine) validated prior to testing was used as the sampling fluid and diluent for all products tested. Application volumes were 1.6 mL of Foam B, 2.0 mL of Rinse E, and 2.1 mL of Gel F, volumes of each indicated to rub dry in 30 s based on testing described above (Table 2). Because the volume of Foam B indicated to dry in 30 s (1.7 mL) could not be achieved with the available pump device, the smaller, conservative volume was used. A log_10_ reduction factor (RF) produced by each product per subject was calculated, and the Hodges-Lehman test was applied for comparison of the mean log_10_ RF to the mean log_10_ RF for the reference procedure, as recommended by EN 1500 rev:2009. In addition, mean log_10_ RFs were compared between the test products using a repeated-measures ANOVA with a random effect for Day (Minitab 16). Follow-up Tukey 95% CIs of the mean log_10_ RF difference between each test product and reference were generated. Test products that demonstrated inferiority (i.e., mean log_10_ RF significantly less than that observed from the reference solution) were classified as not meeting the norm. Non-inferiority of each test product compared to the reference was analyzed as specified by EN 1500 rev:2009; that is, non-inferiority was concluded if all of the Tukey 95% CIs were contained in the interval (-∞, 0.6].

In all statistical tests of differences, a level of 5% (i.e., *P* ≤ 0.05) was used to determine statistical significance. The tests for equivalence amongst product formats were also performed at a significance level of 5%, as explained above. However, to be consistent with EN 1500 rev:2009, the non-inferiority analyses were conducted at a significance level of 2.5%.

## Results

### Influence of ABHR application volume on dry-time

**Figure 1:**
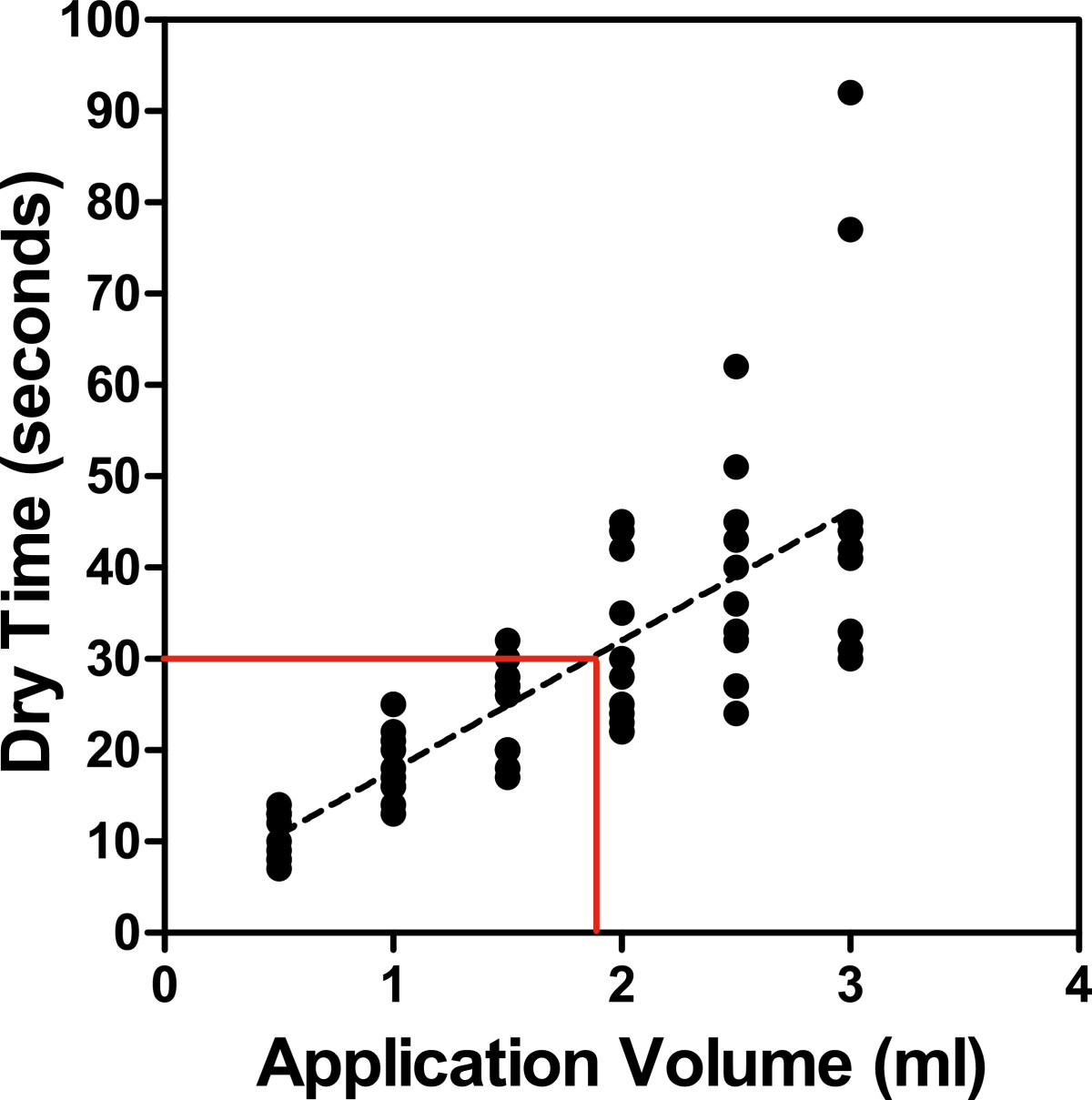
**Relationship between ABHR application volume and dry-time.** Dry-time plotted against ABHR application volume of Gel C. Black circles represent measured dry-times recorded from 11 volunteers at each application volume tested. The black dashed line is the best-fit linear regression, and the solid red line indicates the calculated volume that should rub dry in 30 s.

Dry-times of six ABHR test products, representing different product formats and alcohol concentrations (Table 1), were evaluated at multiple application volumes. Figure 1 illustrates the results for a 70% v/v ethanol gel (Gel C). A significant correlation found between application volume and the time for each test product to dry supported calculation of a rate of drying (s/mL) and established the volume of each product that should dry in 30 s (Table 2). The volumes indicated to dry in 30 s ranged from 1.7 mL (Foam A, Foam B, Rinse D) to 2.1 mL (Gel F). There were significant differences in the rates of drying, with Foam A having the slowest drying rate (18.2 s/mL), and Gel F having the fastest drying rate (12.2 s/mL) (Table 2). Because the test products differed in both alcohol concentration and product format, the influence of each variable on dry-time was explored.

### Influence of product delivery format on ABHR dry-time

To control for other variables, dry-times of three ABHR (Foam B, Gel C, and Rinse D) containing 70% ethanol and nearly identical excipient ingredients were compared (Table 3). Application volumes of 1.6 mL and 0.8 mL were chosen to produce dry-times of approximately 30 s and 15 s, respectively, consistent with recommendations of WHO and CDC [1, 2]. At a 0.8-mL volume, CIs of differences in mean dry-times (see Methods) show that differences in the mean dry-times amongst the three products were no greater than 3.5 s. Thus, the three products yielded statistically equivalent dry-times, on the average. At the 1.6-mL application volume, dripping of Rinse D was observed more frequently (13 of 30 subjects) than of either Gel C (4 of 30 subjects) or Foam A (1 of 30 subjects). Taking product dripping into account, CIs show that differences in the mean dry-times amongst the three products were no greater than 4.9 s. Again, dry-times of the three products were statistically equivalent, on the average.

**Table 3:** **Mean dry-times of three ABHR test products evaluated at two application volumes**

	0.8 mL application volume			1.6 mL application volume		
Test product	Mean dry-time in s^a^	Range in s	# of Subjects dripping product	Mean dry-time in s^a^	Range in s	# of Subjects dripping product
	(95% CI)			(95% CI)		
Foam B	14.7 (13.4-16.1)	7-23	0	28.3 (26.0-30.7)	18-49	1
Gel C	16.0 (14.4-17.7)	10-25	0	25.0 (22.8-27.2)	14-40	4
Rinse D	17.0 (15.4-18.7)	9-25	4	25.0 (22.9-27.1)	15-38	13

^a^ *N* = 30 test subjects; each evaluated all six test configurations in a randomized order.

### Relationship between ABHR dry-time and ethanol concentration

**Figure 2:**
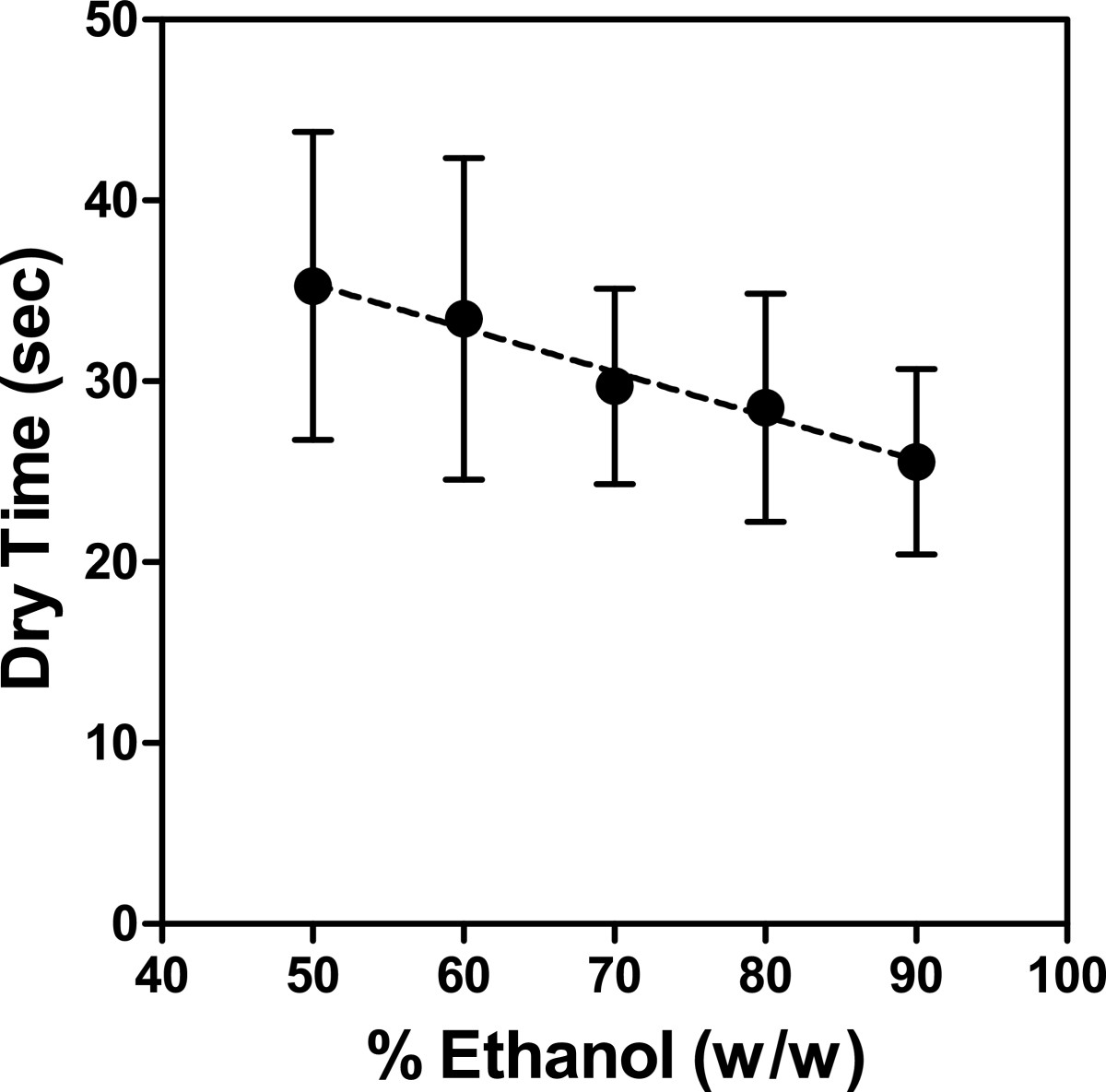
**Relationship between ABHR dry-time and ethanol concentration.** Dry-time for 1.7 mL of various ethanol-in-water rinse solutions plotted against ethanol concentration. Dry-times represent the means of data from 11 volunteers, and error bars represent 95% confidence intervals. The black dashed line is the best-fit linear regression (*Dry Time* = 48.05 - 0.2491(*% Ethanol*).

Figure 2 illustrates that ABHR dry-time decreased linearly with increase in ethanol concentration, a relationship found to be statistically significant (*P* = 0.0035). Furthermore, the mean time to dry for 50% ethanol was significantly greater than that of 80% (*P* = 0.0479) and 90% ethanol (*P* = 0.0015), as was mean dry-time of 60% ethanol versus that of 90% ethanol (*P* = 0.0071).

### Influence of ABHR application volume on antimicrobial efficacy

The efficacies of representative ABHR foam (Foam B), rinse (Rinse E), and gel (Gel F) formulations were evaluated according to the European norm EN 1500 at a standard volume of 3 mL (rubbed for 30 s and rinsed) and, also, at the approximate volumes determined from Table 2 to rub dry in 30 s. The test products, chosen to represent each of the different product formats, contained different alcohol levels and, thus, different volumes drying in 30 s). When evaluated at a volume of 3 mL and rubbed for 30 s, the upper bounds of CIs of mean log_10_ RF differences of each of the three products from the mean log_10_ RF of the reference procedure was less than 0.6 (Table 4). Thus, efficacies of the three test products used at volumes of 3 mL were statistically non-inferior to efficacy of the reference product, thereby satisfying the requirements of EN 1500. Furthermore, efficacies of Foam A, Rinse E, and Gel F were not significantly different from each other when evaluated at 3-mL volumes (*P* = 0.686) or at volumes drying in 30 s (*P* ≥ 0.4292). However, at volumes drying in 30 s, the mean log_10_ RFs produced by the three ABHR were significantly inferior to that of the reference procedure, and thus failed to meet EN 1500 efficacy requirements.

**Table 4:** **Efficacy of three ABHRs evaluated according to procedures of EN 1500**

Test ABHR	Efficacy at 3-mL application volume				Efficacy at volume drying in 30 s			
	Application volume	mean log RF ± SD	*P*-value^a^	CI^b^	Application volume	mean log RF ± SD	*P*-value^a^	CI^b^
EN 1500 reference	2 × 3 mL^c^	4.63 ± 0.60			2 × 3 mL	4.83 ± 0.79		
Foam B	3 mL	4.56 ± 0.68	0.9299	(-0.23, 0.37)	1.6 mL^d^	3.81 ± 0.61	<0.00005	(0.65, 1.38)
Rinse E	3 mL	4.50 ± 0.90	0.6793	(-0.17, 0.43)	2.0 mL	4.03 ± 0.55	<0.00005	(0.44 1.18)
Gel F	3 mL	4.61 ± 0.94	0.9979	(-0.28, 0.32)	2.1 mL	4.02 ± 0.60	<0.00005	(0.44, 1.17)

^a^Comparison of each product with the reference.

^b^95% two-sided CIs on the mean log_10_ RF for each product subtracted from the mean log_10_ RF for the reference. By EN 1500, if the upper confidence limit difference is less than 0.6, then non-inferiority between the test and reference products is established.

^c^Reference product rubbed for a total of 60 s, followed by a 5-s rinse under running tap water.

^d^The volume calculated to dry in 30 s was 1.7 mL. This volume could not be achieved with available pump devices, so a lower (conservative) volume was used.

## Discussion

In this study, we evaluated the influence of several variables on ABHR dry-time, including product format, application volume, and alcohol concentration. Consistent with previous studies, ABHR dry-times increased proportionally to product application volume (Figure 1) [10, 11], and the rates at which different ABHR products dried varied significantly (Table 2). The volume of the two foams evaluated in this study drying in 30 s was 1.7 mL (1.5 g), which is similar to that reported by Kampf *et al*. for ABHR foams (1.6 g) [10]. Furthermore, the drying rates calculated in Table 2 revealed that the two foams were the slowest drying products tested. However, careful examination of Table 2 in the context of alcohol concentration of each test product indicates that rate of drying was faster for products with higher alcohol concentration, regardless of format. Noting the inverse relationship between ABHR dry-time and alcohol concentration clearly demonstrated in Figure 2, the observed differences in test product drying rates likely result from differences in alcohol concentration. That is, as the alcohol concentration of ABHR is increased, the volume of product that will rub dry in 30 s also increases. Note that 2.1 mL of Gel F (90% v/v) dried in 30 s, as did 1.7 mL of Gel A (62% ethanol v/v), a difference of 0.4 mL (Table 2).

By evaluating products of equivalent alcohol concentration, differing only by the presence of thickening or foaming agents, delivery format was found to have no influence on ABHR dry-time (Table 3). This result is not surprising, considering that ABHR formulations consist predominantly of alcohol and water. Despite differences in physical characteristics, Foam B, Gel C, and Rinse D each comprise greater than 97% alcohol and water by weight and differ in composition by less than 3%. This similarity is further evidenced by the identical densities of the 3 products (Table 1). Based on demonstrated influence of alcohol concentration on dry-time (Figure 2) and lack of influence of delivery format on dry-time, we would attribute the differences in drying rates to alcohol concentration.

In agreement with previous studies, product application volume was found to be a critical determinant of ABHR efficacy [4, 7]. Each of the three products evaluated (Foam B, Rinse E, and Gel F) met the performance criteria of EN 1500 when tested at 3-mL application volumes, but failed when tested at volumes drying in 30 s (Table 4). Statistical equivalence of the mean log_10_ RFs was established amongst the three products when applied in volumes that dried in 30 s, despite the fact that Gel F, the test product with the highest alcohol concentration, was also tested at the largest application volume. Taken together, these data suggest that time that product remains wet on the hands may be the most important determinant of ABHR efficacy when evaluated under actual use conditions (i.e., rubbed until dry), irrespective of other variables.

By expanding the studies of Kampf *et al.*[10] to include gels and rinses we can expand upon their conclusions. Regardless of delivery format or alcohol concentration, the volume ABHR drying in 30 s is considerably less than 3 mL. Furthermore, when 3 mL of product are used, dry-times are considerably greater than 30 s. Importantly, when used at volumes drying in 30 s, gel, foam, and rinse products did not meet the efficacy criteria of EN 1500. Therefore, the assertion that the use of ABHR foams would encourage users to apply volumes too low to meet EN 1500 standards [10] should be expanded to include gels and rinses. The data presented herein suggest that it may be more appropriate to make ABHR application recommendations based on formulation specific efficacy data.

Indeed, there is currently limited understanding of the actual ABHR application volumes used by healthcare workers in practice, despite recommendations [1, 2]. A recent survey of ABHR dispensers in U.S. hospitals found that outputs ranged from 0.6 mL to 1.3 mL, and that product dry-times ranged from 12 s to 26 s [16]. Whether healthcare workers use more than one dispenser actuation is unknown; anecdotally, it is expected they most frequently do not. An often-cited publication by Voss and Widmer argued that the use of ABHR saves significant time and can promote hand hygiene compliance [17]. Note, however, that the time estimated for application of an ABHR was 20 s; considerably shorter than the dry-time for the 3-mL volume required to meet EN 1500 efficacy requirements.

To better understand ABHR efficacy under clinical use conditions, “in particular, short application times and volumes”, experts have called for the development of improved *in-vivo* protocols that more closely simulate real-world conditions [2, 18]. EN 1500 specifies that test subjects rub 3 mL of test product onto the hands for 30 s and then to neutralize the remaining alcohol [8]. However, as Table 2 indicates, typical volumes of ABHRs drying in 30 s range from 1.7 to 2.1 mL, and Figure 1 illustrates that 3 mL can take as long as 90 s to dry on the hands. This uncoupling of dry-time from application volume does not represent real use conditions. The modification to EN 1500 used in this study, where the test product is rubbed until dry, with no subsequent rinse, more closely simulates product usage in clinical settings and results in more accurate recommendations for application volume.

Our study has several limitations. For product dry-time experiments, that subjects did not use a standardized rub-in technique may have had a minor impact on dry-times. However, adherence of healthcare workers to the standardized technique is unknown, but unlikely, and this technique has been previously shown to have minimal impact on ABHR efficacy [19]. Efficacy studies were performed using a limited number of subjects and a laboratory-based method with success criteria not linked to clinical outcome, and they should be understood in that way. Lastly, this study evaluated efficacy of only a single representative of each product delivery type (rinse, gel, and foam). Because product formulation also impacts efficacy [15], broad conclusions regarding the efficacy of other products should be made with caution.

## Conclusion

In conclusion, application volume was found to be the primary driver of ABHR dry-time and, therefore, of efficacy. Our data suggest that ABHR application volumes consistent with WHO recommendations may fail to meet EN 1500 efficacy requirements, irrespective of delivery format or alcohol concentration. The implications of this are unknown, but should be of considerable concern. Further research is needed to better understand ABHR application volumes actually used by healthcare workers in clinical practice and how these relate to product effectiveness. Finally, there is a need to develop improved standard test methods that more closely reflect clinical use conditions and provide success criteria linked to clinical outcomes.

## Authors’ original submitted files for images

Below are the links to the authors’ original submitted files for images. Authors’ original file for figure 1 Authors’ original file for figure 2
