# Supplementary figures and images for: The relative influences of product volume, delivery format and alcohol concentration on dry-time and efficacy of alcohol-based hand rubs
Source: BMC Infect Dis. 2014 Sep 20;14:511. doi: 10.1186/1471-2334-14-511 (PMC4180309; doi:10.1186/1471-2334-14-511)

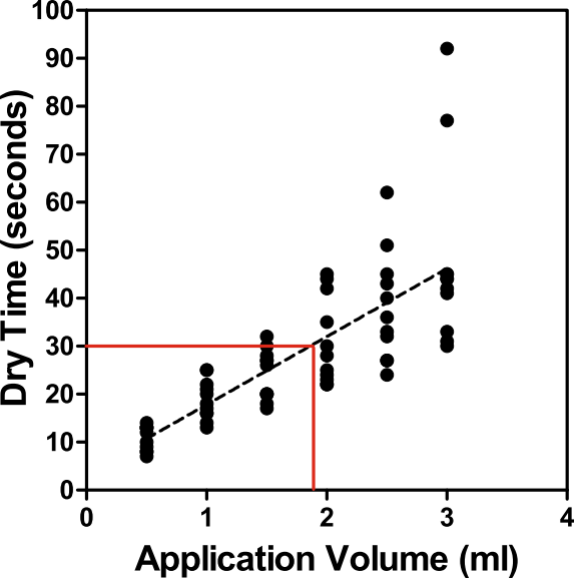

Supplement: Supplementary file 1 — Authors’ original file for figure 1 [file 12879_2014_3830_MOESM1_ESM.pdf]

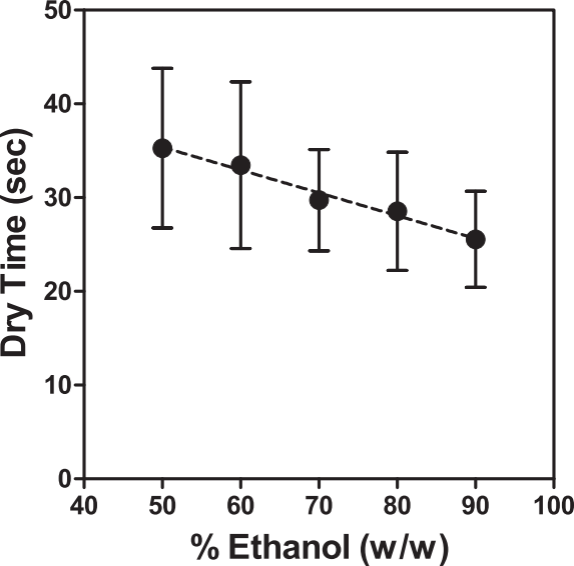

Supplement: Supplementary file 2 — Authors’ original file for figure 2 [file 12879_2014_3830_MOESM2_ESM.pdf]
